# Addressing limitations of the Gait Variability Index to enhance its applicability: The enhanced GVI (EGVI)

**DOI:** 10.1371/journal.pone.0198267

**Published:** 2018-06-01

**Authors:** Arnaud Gouelle, Linda Rennie, David J. Clark, Fabrice Mégrot, Chitralakshmi K. Balasubramanian

**Affiliations:** 1 Gait and Balance Academy, ProtoKinetics, Havertown, Pennsylvania, United States of America; 2 Laboratory « Performance, Santé, Métrologie, Société (PSMS) », UFR STAPS de Reims, Reims, France; 3 Research Department, Sunnaas Rehabilitation Hospital, Nesodden, Norway; 4 Brain Rehabilitation Research Center, North Florida/South Georgia Veterans Health System, Gainesville, Florida, United States of America; 5 Department of Aging and Geriatric Research, University of Florida, Gainesville, Florida, United States of America; 6 Unité Clinique d’Analyse de la Marche et du Mouvement, Centre de Médecine Physique et de Réadaptation pour Enfants Bois-Larris, Lamorlaye, France; 7 Biomécanique et Bioingénierie UMR CNRS 7338, Sorbonne Universités, Université de Technologie de Compiègne (UTC), Compiègne, France; 8 Department of Clinical and Applied Movement Sciences, University of North Florida, Jacksonville, Florida, United States of America; University of Malaya, MALAYSIA

## Abstract

Prior research has established the Gait Variability Index (GVI) as a composite measure of gait variability, based on spatiotemporal parameters, that is associated with functional outcomes. However, under certain circumstances the magnitude and directional specificity of the GVI is adversely affected by shortcomings in the calculation method. Here we present an enhanced gait variability index (EGVI) that addresses those shortcomings and improves the utility of the measure. The EGVI was further enhanced by removing some input spatiotemporal variables that captured overlapping/redundant information. The EGVI was used to reanalyze data from four previously published studies that used the original GVI. After removing data affected by the GVI’s prior shortcomings, the association between EGVI and GVI values was stronger for the pooled dataset (r^2^ = 0.95) and for the individual studies (r^2^ = 0.88–0.98). The EGVI also revealed stronger associations between the index value and functional outcomes for some studies. The EGVI successfully addresses shortcomings in the GVI calculation that affected magnitude and directional specificity of the index. We have confirmed the validity of prior published work that used the original GVI, while also demonstrating even stronger results when these prior data were re-analyzed with the EGVI. We recommend that future research should use the EGVI as a composite measure of gait variability.

## Introduction

The Gait Variability Index, GVI [1], is a generic, conglomerate measure that objectively quantifies the variability measured in spatiotemporal variables during gait. Prior to the development of GVI, assessment of gait variability was shown to be limited by the methodology used. First, it is unclear which spatiotemporal measures are of greatest importance when assessing gait variability. Variability has been reported for at least 11 spatiotemporal parameters [1], but it is uncertain as to which are the most relevant in relation to mobility or cognitive functions and the subsequent deficits that these parameters reflect. Other issues to consider in relation to gait variability is how to deal with interdependence between variables. For instance, if one should use stride-to-stride variability or step-to-step variability, spatial versus temporal variability. Lastly it is not clear if variability should be considered as a global parameter or should the two lower limbs be considered separately [2]. Gait variability is typically reported as the within subject standard deviation or coefficient of variation for multiple steps or strides. However, standard deviation is sensitive to the scale and the coefficient of variation tends toward high values when the mean is close to 0. In addition, reliable gait variability measures require multiple steps and strides to minimize measurement error [3]. Most often, data from several walking bouts are combined as if it was only one long walking trial. In this situation, the amount of gait variability is artificially increased by inter-trial variability. The development of a conglomerate measure was reported to solve these methodological challenges [4]. Furthermore, the GVI was shown to correlate with clinical outcomes of gait and balance in 12-to-25-year-old individuals with Friedreich’s ataxia [1], was sensitive in capturing the gait variability changes from childhood through adulthood [5] and demonstrated validity in individuals with mobility deficits [4, 6–10]. Therefore, mounting literature supported the use of the GVI as a valid outcome measure of gait variability.

The GVI quantifies the distance between the amount of variability in a reference group and the amount of variability in an individual. The natural logarithm of this raw distance is then used to transform the value of the GVI into a score, which is based on the number of SDs separating the individual from the reference group, where 100 and 10 respectively represent the mean and SD of the score of the reference group. While the GVI seems to solve most of methodological problems surrounding other gait variability measures, there are some unresolved issues. These were highlighted in a recent article by Rennie et al. [11] where it was shown that the GVI did not display known increases in gait variability for individuals with Parkinson’s disease as compared to controls, even though most of the ‘spatiotemporal parameters *p*_*n*_’ [1] used in the GVI calculation were significantly different from control values. Also, the GVI was not able to discriminate individuals with mild from moderate Parkinson’s disease. We believe that two limitations of the GVI may have led to these results. The first limitation, referred to as “the magnitude problem”, arises when the raw distance between the individual and reference group approaches zero. When this occurs, the function of the natural logarithm generates an artificially high z-score that results in GVI scores higher than 100. Therefore, if GVI<100 indicates abnormal variability as with impaired gait, a GVI≥100 indicates that the patient has a similar level of variability as the healthy population (neither too little nor too much), and there is no interpretable difference between a GVI of 100, 115 or 130. This magnitude problem is especially an issue for research purposes when comparative statistics are used to analyze data sets that include GVI values above 100. The second limitation, referred to as “direction specificity problem”, relates to the possibility of obtaining the same GVI score (e.g. 90) because of both high variability or low gait variability. The GVI is not designed to make this distinction between high and low variability because it is computed as the absolute distance between an individual’s variability compared to the reference mean variability, independent of the direction. This lack of direction specificity may mask any changes on the continuum of gait variability.

Therefore, the purpose of this study was to propose an enhanced version of the GVI, the EGVI. The EGVI was developed to resolve the limitations with the GVI and further optimized by excluding some overlapping/redundant spatiotemporal variables. Furthermore, published data from four previous articles that used the GVI [1–5,11] were compared to the EGVI. We hypothesized that the EGVI would eliminate the methodological problems of the GVI without altering the results from already published data.

## Methods

### Solving the magnitude problem (Fig 1)

When the absolute distance “(*d*^*⍺*,*HP*^)” between an individual “α”and reference “HP” (healthy population) is obtained, the natural logarithm of this value is computed (*raw GVI* = ln (*d*^α,*HP*^)) to get a raw index [1]. As the function *ln*(*x*) moves toward negative infinity for a value of x close to 0 and as *ln*(1) = 0, the resulting values of ln(*d*^α,*HP*^) for *d*^*⍺*,*HP*^ within the range [-1;0] tends to become increasingly more negative when the distance *d*^*⍺*,*HP*^ is very small (i.e., when there is similar variability for the individual and reference group). Next, the z-score is calculated, i.e. the number of SDs separating the raw score of an individual from the raw score of the reference group. As the reference mean and SD of the raw GVI are respectively 1.61 and 0.91, the z-score also increases artificially. For example, if an individual α is very close to the reference with a distance *d*^*⍺*,*HP*^ of 0.01, then ln (0.01) will be -4.61. Once the z-score is computed, the individual will be at 6.84 SDs from the reference, as shown below:

$$raw\;GVI\;of\;individual\;\alpha =\ln{(d}^{⍺,HP})=ln\;(0.01)=-4.61$$

zscoreforGVI=(rawGVIofindividualα–meanrawGVIinreference)/(SDrawGVIinreference)=(−4.61−1.61)/0.91=6.84.

**Fig 1:**
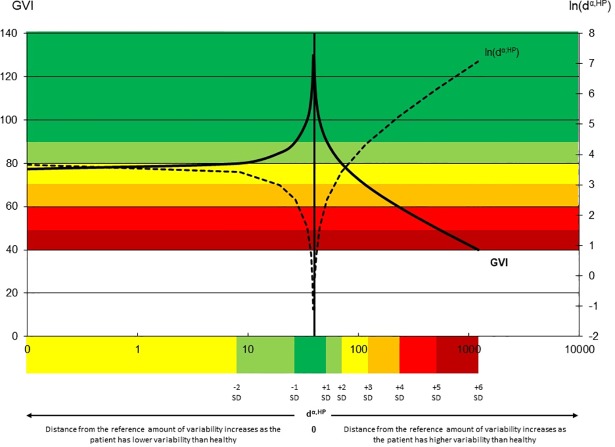
Representation of the values for the original GVI (left axis) and the raw GVI, i.e. = ln(d^⍺^,HP) (right axis), in function of the distance d^⍺^,HP between the patient and the reference population (horizontal axis, logarithm scale). A zero distance from the HP is represented by the vertical line. The more the distance increases the more the raw GVI increases leading to a decreasing GVI score. Towards left when the patient is less variable than HP, towards right when the patient is more variable. The colors in the horizontal axis show the limits for 1 standard deviation separating the patient and the HP. The colors in the graphic show the GVI obtained for these ranges. This figure illustrates both the magnitude problem and the possibility to get a same GVI with higher and lower variability than HP.

After the final transformation, where 1 z-score equals 10 points of separation from 100, the GVI for individual α is 168.

To avoid artificially high z-scores, for the computation of the EGVI, we added +1 to the computed distance *d*^*⍺*,*HP*^. In other words, if the individual α has a distance of 0.01 (previous example), the new distance will be 1.01 and so *ln* (1.01) = 0.01.

### Solving the lack of directional specificity

The absolute difference in gait variability between an individual and the reference group, whether positive or negative, will produce a single GVI value. It is important for the EGVI to be able to differentiate these two situations.

Therefore, we considered that the EGVI is 100 if the raw GVI for the individual is comprised within [- mean raw EGVI in controls; + mean raw EGVI in controls]. EGVI = 100 indicates that the person has a similar level of variability as the reference (neither too little nor too much). Each 10-point difference corresponds to a separation of one SD from the reference, indicating that the variability of the individual is greater than (EGVI >100) or lower (EGVI <100) than the amount of variability observed in normal gait.

### Other optimizations

The GVI is the mean of the left and right side GVI computed from spatiotemporal parameters related to one side (left or right limb). To enhance the capability of the EGVI in differentiating variability between legs, some optimizations were performed. Of the 9 previous variables, 5 were retained. The retained spatiotemporal variables in the EGVI are step length (cm), step time (s), stance time (s), single support time (s), and stride velocity (cm/s). Stride length and stride time were considered redundant in relation to step length and step time. Also, they were removed because they include simultaneous information from left and right sides. We also removed swing time since it is the same as the contralateral single support time.

### Computing and validating the EGVI using secondary data

Once all optimizations were performed, data from four previous publications using the original version of the GVI was extracted to compute the EGVI scores, and the results were compared.

When GVI<100, the gait variability is increased or decreased compared to the reference and GVI≥100 indicates a normal amount of gait variability. The EGVI differentiates low and high variability, and EGVI<100 indicates low variability, EGVI = 100 similar amount of variability as the reference group, EGVI>100 high variability.

To compare unbiased values of the GVI scores to the EGVI, data that corresponded to GVI>100 (magnitude problem), or GVI<100 and EGVI<100 (directional problem) were removed before calculating the coefficients of determination. Previously published data for individuals with Friedreich ataxia (FRDA dataset) [1], typically developing children (TD dataset) [5], older adults (OA dataset) [4], and for individuals with mild to moderate Parkinson’s Disease (PD dataset) [11] were re-analyzed using the same statistics as in the original publications (more details about statistics can be found in Figure A and Table A in S1 Appendix). For the FRDA dataset, Pearson’s correlations were used to investigate relationships between EGVI and FAPS, 8 m walk test time, Lower limb testing, ICARS, and PGD subscale [see Table A1 in S1 Appendix for details]. In the TD dataset, non-parametric rank tests (Spearman’s r) were carried out on the data of the 140 children and teenagers to evaluate the relationships between the EGVI and other spatiotemporal gait parameters. In the OA dataset, Pearson correlation coefficients investigated the relationship between EGVI and clinical measures of functional mobility and balance, including number of falls in the past year, Berg Balance Scale, Short Physical Performance Battery, Activities-Specific Balance Confidence, Timed Up and Go Test, Community Balance and Mobility Scale, Dynamic Gait Index and Functional Reach Test. For the PD dataset, the Pearson’s correlation coefficient was used to investigate the association between EGVI and the Mini-BESTest and the TUG. Due to a heteroscedastic distribution in the data, an inverse transformation was performed on the TUG scores (1/TUG). Like for the GVI in the article by Rennie et al. [11], the responsiveness of the EGVI was examined by ROC curve statistics to explore to what extent the index was able to discriminate between individuals classified Hoehn & Yahr 2 and 3. The main clinical feature that separates H&Y 2 and 3 is the manifestation of postural instability in those classified as H&Y 3 as opposed to 2. It was therefore hypothesized that those graded as H&Y 3 would have higher EGVI scores as decreasing balance capabilities are associated with higher gait variability [12, 13].

## Results

Table 1 presents the data re-analyzed to calculate the EGVI. Combining data from the 4 data sets resulted in a total of 402 participants. For all data, the r^2^ between GVI and EGVI was 0.87. When the data affected by the shortcomings of the GVI were removed, the total data set reduced to 320 participants and the r^2^ between GVI and EGVI increased to 0.95.

**Table 1:** Description of data sets re-analyzed in the current study.

REF	POPULATION	GROUPS	N	GVI SCORES AFFECTED BY		GVI (after excluding data with problem)		REVISED EGVI (all subjects)				
				Magnitude problem	Lack of direction	Mean (SD)	Range	Mean (SD)	Range	EGVI <100	EGVI = 100	EGVI >100
[1]	Patients (aged 12–25) with Friedreich ataxia, classified by Posture and Gait Disturbances ICARS subscale	All assessments	81	0	0	70.4 (8.0)	51–90	139.9 (11.8)	105–169	0	0	81
		PGD score [1–9]	17	0	0	75.6 (6.1)	68–89	131.6 (9.4)	114–144	0	0	17
		PGD score [10–17]	48	0	0	71.3 (6.9)	57–90	139.0 (10.2)	105–160	0	0	48
		PGD score [18–25]	16	0	0	61.9 (5.4)	51–74	153.0 (9.5)	130–169	0	0	16
[5]	Typically developing children from 1 to 17 years old, categorized into 7 groups of 20 based on age	≤ 3 years	20	0	0	70.8 (7.3)	46–80	136.0 (11.6)	122–175	0	0	20
		4–5 years	20	0	0	77.0 (5.3)	69–87	126.2 (8.6)	108–137	0	0	20
		6–7 years	20	0	0	79.8 (3.9)	73–87	122.8 (6.2)	111–135	0	0	20
		8–9 years	20	0	0	82.5 (5.3)	72–92	118.6 (8.2)	105–135	0	0	20
		10–11 years	20	0	0	87.1 (4.7)	80–97	114.2 (6.4)	102–123	0	0	20
		12–13 years	20	3	0	**89.9 (4.1)**	**83–99**	109.6 (5.6)	100–120	1	2	17
		14–17 years	20	4	1	**93.6 (5.0)**	**85–100**	105.1 (6.0)	99–121	1	5	14
[4]	Older adults from 65 to 90 years old, categorized into high functioning (HFOA) or mobility deficits older adults (MDOA)	All data pooled	81	11	10	**88.1 (7.4)**	**71–100**	110.0 (11.4)	90–138	13	12	56
		[OA]–Study 2	19	2	1	**86.3 (6.5)**	**76–98**	113.7 (11.7)	90–133	2	3	14
		[OA]–Study 3	34	4	9	**91.2 (6.8)**	**73–100**	105.1 (10.1)	90–135	11	5	18
		[HFOA]–Study 4	15	4	0	**91.2 (5.3)**	**84–98**	106.7 (6.0)	100–117	0	4	11
		[MDOA]–Study 5	13	1	0	**82.9 (7.7)**	**71–100**	120.8 (10.3)	104–138	0	0	13
[3]	Subjects with idiopathic PD, with mild to moderate PD and ≥ 60 years	All cohort	100	44	9	**87.7 (7.2)**	**76–99**	107.1 (10.3)	91–132	12	35	53
		Hoehn & Yahr 2	44	22	8	**86.4 (4.9)**	**79–97**	105.0 (9.1)	91–125	8	14	22
		Hoehn & Yahr 3	56	22	1	**88.2 (7.9)**	**76–99**	108.7 (11.0)	97–132	4	21	31

The data in bold are different from the results published previously due to the presence of GVI presenting magnitude or lack of direction problem.

### FRDA dataset

**Fig 2:**
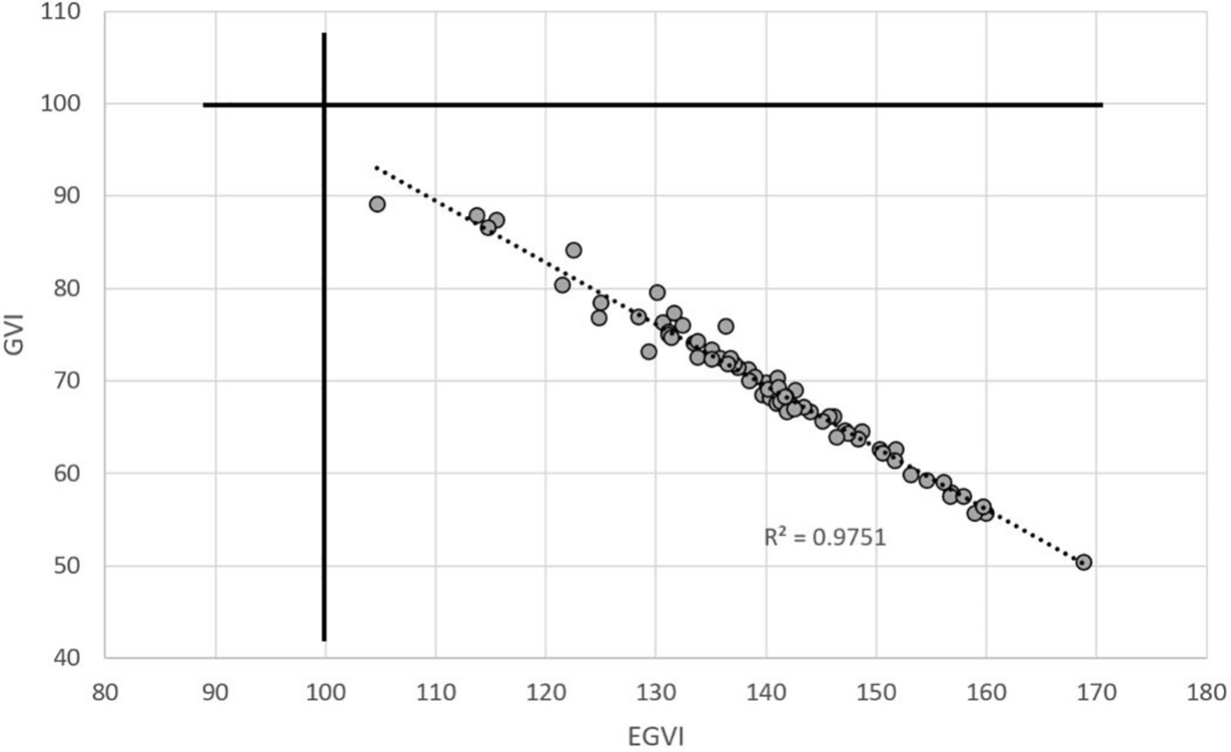
Relationships between GVI (vertical axis) and EGVI, in the data from Gouelle et al. (2013).

Re-analyzing the dataset resulted in EGVI of 139.9 ±11.8, while it was 70.4 ±8.0 for the GVI. All individuals had an EGVI>100. Therefore, the r^2^ between GVI and EGVI for this data set was 0.98 (Fig 2) and statistics were similar to the published results.

### TD dataset

**Fig 3:**
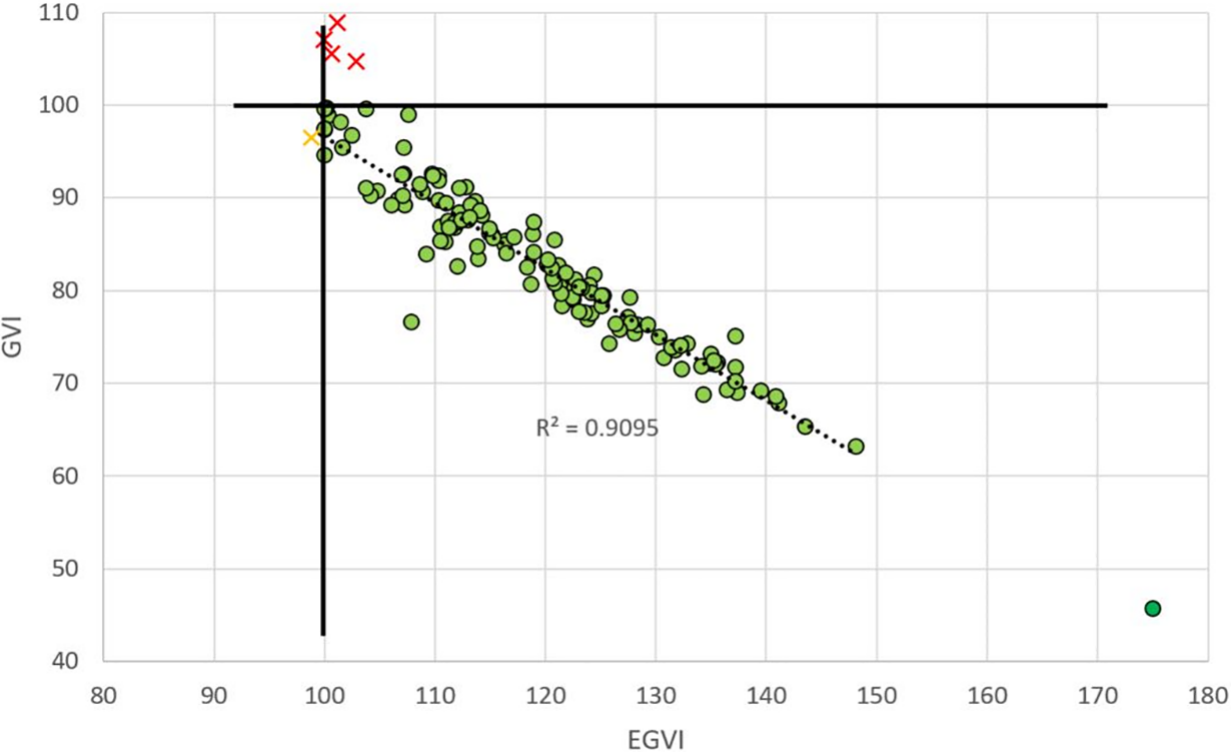
Relationships between GVI (vertical axis) and EGVI, in the data from Gouelle et al. (2015). The point in dark green represents the younger child who walked independently only for two weeks and is provided to give an idea about what could be about the ceiling of EGVI (175) for a high level of unsteadiness in ambulation.

All the children were more variable than adults, except seven individuals in the two older groups (12–13 and 14–17 years-old) who had GVI higher than 100 due to a smaller distance *d*^*⍺*,*HP*^ than the reference population. Only one adolescent in the 14–17 years-old group showed both GVI and EGVI<100 (96.4 and 98.9 respectively). The r^2^ between GVI and EGVI was 0.88 and increased to 0.91 once the affected data were removed (Fig 3).

### OA dataset

**Fig 4:**
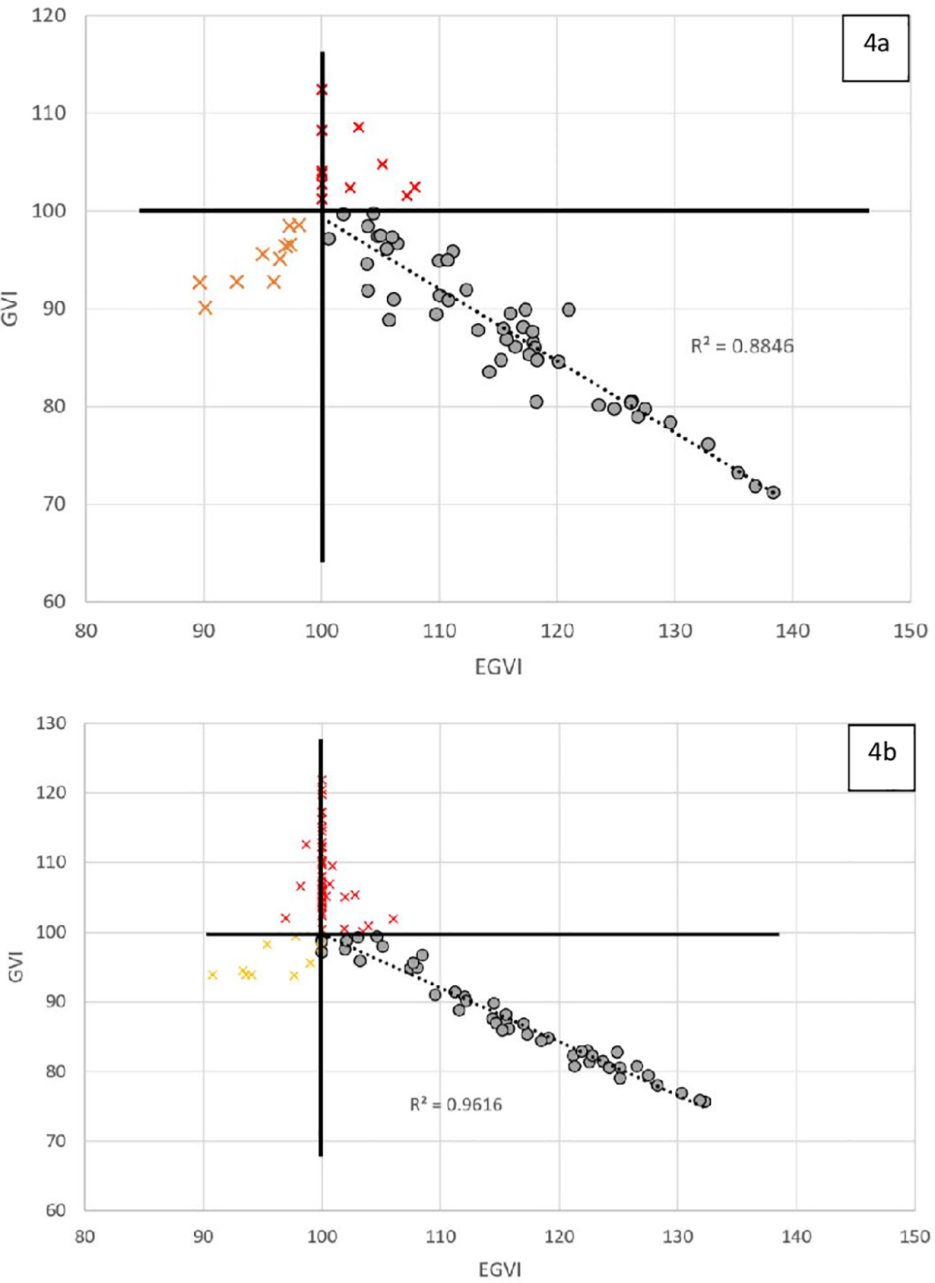
**Relationships between GVI (vertical axis) and EGVI, in the data from Balasubramanian et al. (2016) (4a) and in the data from Rennie et al. (2017) (4b).** The coefficient of determination was computed once all data represented by crosses were removed. The crosses in the area where GVI<100 and EGVI<100 correspond to data for which the lower GVI was due to lower variability than HP. The crosses for GVI>100 are from individuals whose distance *d*^*⍺*,*HP*^ was smaller than mean HP.

11 individuals had GVI scores >100 reflecting the magnitude problem (Fig 4A—red crosses). The outcomes were also affected by 10 persons presenting GVI<100 (90.1 to 98.6), while they were less variable than the normal reference (EGVI>100) (Fig 4A—orange crosses). Once these ‘false positive’ values and GVI>100 were removed, r^2^ between GVI and EGVI was 0.88 (the r^2^ was 0.67 when affected data were included).

Importantly, while the GVI correlated only with Berg Balance Scale, the EGVI significantly correlated, at moderate level (r between 0.5 and 0.7 [14]), with all the study clinical measures of balance and mobility performance, and weakly correlated with falls history (see Table A3 in S1 Appendix for details).

### PD dataset

Fig 4B shows the GVI scores of the PD cohort studied by Rennie and colleagues. These PD participants were the most impacted by the magnitude problem (n = 44, marked as red crosses) and lack of directional specificity (n = 9, marked as yellow crosses). With these cases removed, the r^2^ between the GVI and the EGVI was 0.96, while the r^2^ was 0.70 when all data were considered.

The GVI and EGVI values for the PD cohort are summarized in Table A4. Out of the 100 subjects, 53 were identified as having increased gait variability with a mean overall EGVI of 114.2 (9.4) and 116.3 (9.1) for the most affected side only. Further, 12 individuals were identified as having lower gait variability than the reference mean, with a EGVI of 96.3 (2.8) (overall) and 94.7 (3.5) (most affected). No significant differences were found between EGVI scores for H&Y 2 and 3.

The correlation between the EGVI and the MiniBESTest and TUG improved as compared to the GVI, however, they were still low to moderate (see Table A5). Lastly, Table A6 shows the comparative result of the ROC analysis, showing the ability of the GVI and the EGVI to discriminate mild from moderate disease severity in PD. The results show low discriminatory ability in both indices.

## Discussion

The GVI has served as a quantifiable conglomerate index of gait variability and has been validated in healthy controls and those with impaired mobility. However, some issues remain with the GVI; artificially high scores (i.e., magnitude problem) and the lack of ability to distinguish high and low variability (i.e., lack of direction specificity). The purpose of this study was to propose an improved version of the GVI, the enhanced gait variability index. We hypothesized that the EGVI would solve the magnitude problem and lack of direction specificity without compromising the final outcome measure. The results of our study were in agreement with our hypothesis. When analyzing previously published datasets, the results were similar and, when problematic data where present, they were better using the EGVI.

In populations with higher variability than heathy adults (like in FRDA or TD datasets), the GVI is not affected by either of the issues of magnitude or lack of direction specificity because the subjects are all more variable than the healthy controls. Specifically, the FRDA data set included individuals with Friedreich’s ataxia, a condition where motor control is severely compromised, and it is expected that individuals with Friedrich’s ataxia have higher gait variability. Similarly, typically developing children are expected to have gait patterns that are different from adults. Therefore, the results for the FRDA and TD datasets did not change from previously published data and in addition, the correlation between the existing version of the GVI and the EGVI was high. Nonetheless, the additional improvements added to the EGVI enables standardized reporting in these datasets and likely better differentiation.

The issues with the original version of the GVI, in turn, are more apparent when individuals within a population have an amount of variability close to the healthy population, as in the OA and PD datasets, or are even less variable. The OA dataset included older adults (aged 65 years and older) across a range of mobility limitations, from low to high functioning. Therefore, it can be expected that this dataset included some individuals with variability similar to the healthy reference population, leading to uninterpretable GVI above 100, that turn into 100 when EGVI is used (i.e., normal variability). In addition, specifying direction with the EGVI allowed for identification of those older adults with lower variability than the reference group. A minimum level is required to ensure ability to regulate step-to-step variations [15] and a lower level might demonstrate a too-rigid walking pattern. Importantly, the EGVI led to stronger associations with clinical assessments of mobility function in the OA dataset. While previously published results concluded that the GVI demonstrated good validity in the older adult population based on the correlation of the GVI with some gait and mobility outcomes, the EGVI correlated moderately with all gait and mobility outcomes included in this study strengthening the validity of the EGVI for use in this population. The relationship between number of falls in the past year and EGVI demonstrated better correlation than between GVI and history of falls, however the association was statistically weak. The type of falls reported for this sub-set of the study included several unusual falls in high-level functions like running and sporting activities. Falls were also recorded retrospectively [4]. Prospective reporting of fall events is a more accurate approach and minimizes recall bias. However, despite these concerns with the falls recording, the trend for an association between EGVI and falls history is encouraging. Future studies should replicate these analyses with prospective falls data collection. In addition, since gait variability has been demonstrated to be a biomarker of falls risk in various clinical populations [16], a global outcome representing gait variability such as the EGVI will assist to standardize variability measurement approaches. Relationship with falls has also to be analyzed in light of the mean spatiotemporal parameters, because compensative strategies can be used by the patient to limit the instability and/or to facilitate the control of balance regulation [2].

The calculated EGVI values for the PD data set demonstrates how the method differentiates between those individuals with higher, lower and normal gait variability (see Table A4 in S1 Appendix), and the proportions in which the level of gait variability can vary within this population. The results show that 65% of the PD cohort was identified as having altered levels of gait variability as compared to the reference population, where 53% of the subjects had higher gait variability and 12% had lower. It is not unlikely that those individuals with PD that are more affected by rigidity could display a more inflexible gait pattern, whereas those with decreased functional balance and mobility could have a more varied gait pattern from one step to the next. This diversity also explains why the EGVI mean for the total group was only 107 (Table 1), giving the somewhat wrongful impression that the PD cohort displayed only slightly elevated levels of gait variability. It was therefore interesting to consider those with higher gait variability separately, showing a sub-group mean overall EGVI of 114.2, and 116.9 for the most affected side only, corresponding to approximately 1.5 standard deviations from the reference mean. However, when comparing these values to the mean EGVI estimated for the healthy older adults in the OA data set, the EGVIs for the two groups seem similar, suggesting comparable levels of gait variability. This finding is in contrast to previous studies reporting higher levels of gait variability in separate spatiotemporal variables for individuals with PD as compared to healthy controls and mirrors previous comparisons between the groups with the GVI [11].

Further, it could be hypothesized that those in the higher gait variability sub-group would show improved association with functional balance and mobility scores with the EGVI, than those previously reported by Rennie et al. [11] for the GVI. Interestingly, the EGVI showed strengthened correlations with functional balance which were moderate, however, this was not the case with regards to mobility as represented by the Timed Up and Go test (Table A5 in S1 Appendix). The EGVI is designed to assess the variability during straight walking, while the TUG assesses several components (sit-to-stand, walking and turning) in a same performance score. A better performance in gait variability might be counter-balanced by persisting deficits during the turning phase of the TUG. This could be a possible explanation as to why the association between gait variability and TUG may remain low despite the improvements made to the EGVI.

Lastly, the subjects included in the PD data set were individuals with mild to moderate disease severity where the main difference between those classified as H&Y2 and 3 is reduced postural responses for H&Y3. The ability of the EGVI to distinguish between those classified as mild as compared to moderate was not significantly improved from the GVI. This was also true when only those with increased gait variability were considered (see Table A6 in S1 Appendix). Further investigation into the validity of using the EGVI on gait data from individuals with PD is warranted.

In addition to fixing the two issues of magnitude and direction, the EGVI included other optimizations. The spatiotemporal parameters that included variability from both legs (i.e., stride time, stride length and double support time) were deleted to enhance the identification of inter-limb variability. Interrelated spatiotemporal parameters (i.e., swing time) were also deleted to avoid redundancy. This reduction in number of spatiotemporal parameters included in the calculation of the EGVI did not affect the outcome.

Our study had some limitations. It is plausible that the sensitivity in the calculation of the EGVI may have been improved using coefficients from a specific population. However, we validated the EGVI on varied datasets. Future work should establish the reliability of the EGVI and further test the ability of this enhanced version to discriminate inter-limb variability.

Clinical evaluation of gait typically focuses on visible impairments such as slow speed and asymmetry. Mounting evidence suggests that gait variability is a relevant outcome measure reflecting gait and mobility deficits that may be otherwise masked by average measures [17]. Importantly, gait variability is suggested to be a biomarker of fall risk. Despite these suggestions in the literature, gait variability is seldom used as an outcome in clinical settings. One of the reason for the lack of clinical translation might be the challenges with the measurement approaches. EGVI, a global parameter of gait variability, is a relatively simple approach to evaluate variability that solves these measurement challenges. Therefore, the EGVI assists to standardize gait variability measurement approaches and can enhance translation to clinical settings.

## Conclusion

The calculations for the GVI had shortcomings under certain circumstances, which we have resolved with this enhanced gait variability index (EGVI). We have confirmed the validity of prior published work that used the original GVI, while also demonstrating even stronger results when these prior data were re-analyzed with the EGVI. We recommend that future research should use the EGVI as a composite measure of gait variability.

## Supporting information

S1 Appendix Comparative results for the statistical tests done in the published papers presenting GVI scores against the Enhanced Gait Variability Index (EGVI). **Table A1. Pearson’s correlations with the GVI and the EGVI. Table A2. Spearman’s correlations with the GVI and the EGVI. Table A3. Spearman’s correlations with the GVI and the EGVI. Table A4. EGVI in individuals with Parkinson’s Disease (means and standard deviations). Table A5. Associations between dynamic balance and mobility and the GVI and the EGVI. Table A6. ROC analysis: Area Under the Curve for GVI and EGVI**^**(high)**^**. Figure A1. Means and 95% CI for EGVI according to the ‘‘Posture and Gait Disturbances” ICARS sub-score. Figure A2. Means and 95% CI of EGVI for each age group.** The position of the data on the x-axis corresponds to the mean age of the subjects for the considered group (e.g., the data for the ≤3-year-old group are at 2.5 years). **Figure A3. Sensitivity and specificity of the overall EGVI(high) (A) and the most affected side EGVI(high) (B).** The EGVI for those with higher gait variability than the reference mean is included in the analysis (n = 53).
(DOCX)
