# Supplementary material for: Addressing limitations of the Gait Variability Index to enhance its applicability: The enhanced GVI (EGVI)
Source: PLoS One. 2018 Jun 1;13(6):e0198267. doi: 10.1371/journal.pone.0198267 (PMC5983480; doi:10.1371/journal.pone.0198267)
Supplement: S1 Appendix — Table A1. Pearson’s correlations with the GVI and the EGVI. Table A2. Spearman’s correlations with the GVI and the EGVI. Table A3. Spearman’s correlations with the GVI and the EGVI. Table A4. EGVI in individuals with Parkinson’s Disease (means and standard deviations). Table A5. Associations between dynamic balance and mobility and the GVI and the EGVI. Table A6. ROC analysis: Area Under the Curve for GVI and EGVI(high). Figure A1. Means and 95% CI for EGVI according to the ‘‘Posture and Gait Disturbances” ICARS sub-score. Figure A2. Means and 95% CI of EGVI for each age group. The position of the data on the x-axis corresponds to the mean age of the subjects for the considered group (e.g., the data for the ≤3-year-old group are at 2.5 years). Figure A3. Sensitivity and specificity of the overall EGVI(high) (A) and the most affected side EGVI(high) (B). The EGVI for those with higher gait variability than the reference mean is included in the analysis (n = 53). (DOCX) [file pone.0198267.s001.docx]

Supporting Information

**Appendix S1**

This supplementary file presents comparative results for the statistical tests done in the published papers presenting GVI scores against the Enhanced Gait Variability Index (EGVI). For example, if correlation between GVI and one clinical assessment had been done, we performed the same statistical test with EGVI.

Page 2: Friedreich ataxia

Page 3: Typically-developed children

Page 4: Old adults

Pages 5-6: Parkinson’s disease

**FRIEDREICH ATAXIA DATASET**

In the original article [1], Pearson’s correlations were used to investigate relationships between GVI, ICARS and clinical results in the patient group. An alpha-level of 0.05 was considered as significant. To assess the behavior of GVI with regard to the degree of balance and gait impairment, patients were categorized into three sub-groups according to their PGD score. ANOVA and post hoc tests (Fisher’s LSD) were carried out to identify differences between sub-groups.

Correlations are presented below. Like GVI, the EGVI scores were significantly different between all groups.

**Table A1.**

Pearson’s correlations with the GVI and the EGVI.

|  | GVI | | | EGVI | | |
| --- | --- | --- | --- | --- | --- | --- |
| Measures | Pearson’s r | r² | Threshold p< | Pearson’s r | r² | Threshold p< |
| FAPS | 0.56 | 0.32 | 0.01 | -0.47 | 0.22 | 0.01 |
| 8 m walk test time | -0.57 | 0.33 | 0.01 | 0.56 | 0.31 | 0.01 |
| Lower limb testing | 0.24 | 0.06 | 0.05 | -0.26 | 0.07 | 0.05 |
| ICARS | 0.54 | 0.29 | 0.01 | 0.52 | 0.27 | 0.01 |
| PGD subscale | -0.68 | 0.46 | 0.01 | 0.62 | 0.38 | 0.01 |


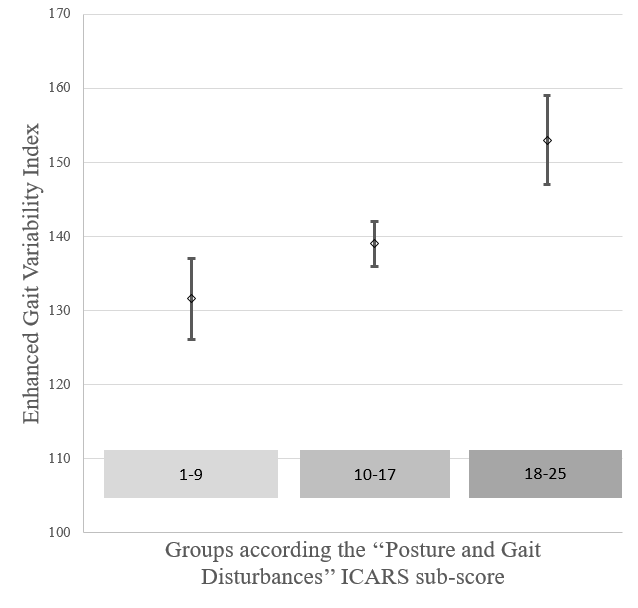


**Figure A1.** Means and 95% CI for EGVI according to the ‘‘Posture and Gait Disturbances’’ ICARS sub-score.

**TYPICALLY-DEVELOPED CHILDREN DATASET**

In the original article [5], non-parametric rank tests (Spearman’s r) were carried out on the data of the 140 children and teenagers to evaluate the relationships between the GVI and other parameters.

The Mann-Whitney U tests showed significant differences between all the pairs of groups (p < .05), except between the 4–5 and 6–7-year-old groups and between the 6–7 and 8–9-year-old groups.

**Table A2.**

Spearman’s correlations with the GVI and the EGVI.

|  | Correlation with GVI | | Correlation with EGVI | |
| --- | --- | --- | --- | --- |
|  | Spearman’s r | p< | Spearman’s r | p< |
| Age (years) | 0.87 | 0.01 | -0.82 | 0.01 |
| Height (m) | 0.82 | 0.01 | -0.78 | 0.01 |
|  |  |  |  |  |
| *Raw parameters* |  |  |  |  |
| Step length (cm) | 0.83 | 0.01 | -0.81 | 0.01 |
| Base of support (cm) | 0.00 |  | 0.02 | n.s. |
| Cadence (steps/min) | -0.59 | 0.01 | 0.53 | 0.01 |
| Velocity (cm/s) | 0.60 | 0.01 | -0.64 | 0.01 |
|  |  |  |  |  |
| *Normalized parameters* |  |  |  |  |
| Step length | -0.09 |  | 0.05 | n.s. |
| Base of support | -0.57 | 0.01 | 0.53 | 0.01 |
| Cadence | 0.19 |  | -0.24 | n.s. |
| Velocity | 0.02 |  | -0.08 | n.s. |

n.s. non-significant


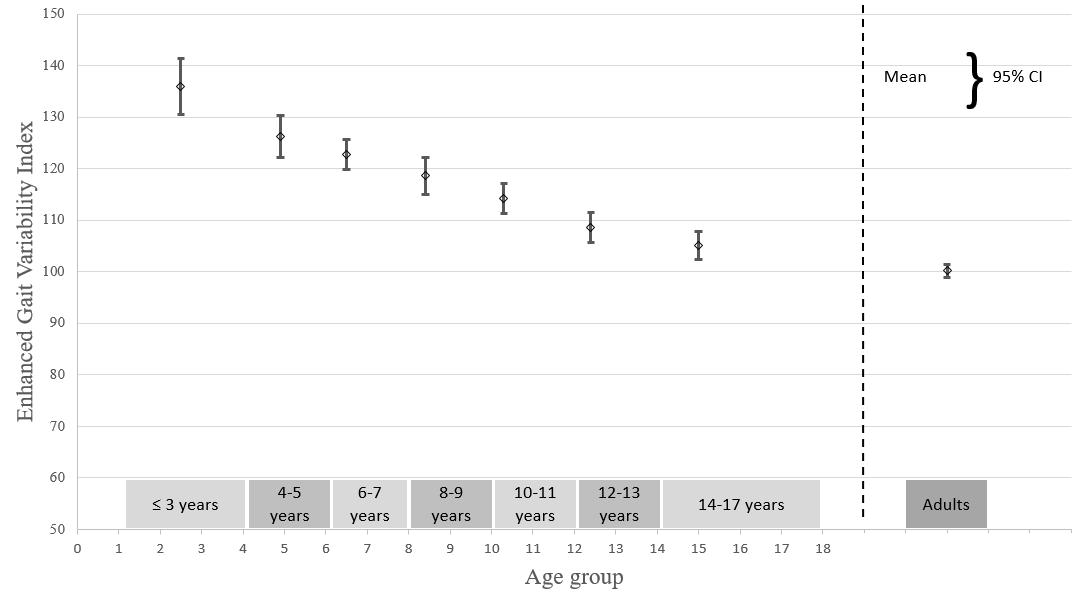


**Figure A2.** Means and 95% CI of EGVI for each age group. The position of the data on the x-axis corresponds to the mean age of the subjects for the considered group (e.g., the data for the ≤3-year-old group are at 2.5 years).

**OLD ADULTS DATASET**

Pearson correlation coefficients investigated the relationship between EGVI and clinical measures of functional mobility and balance.

**Table A3.**

Spearman’s correlations with the GVI and the EGVI.

|  | Correlation with GVI | | Correlation with EGVI | |
| --- | --- | --- | --- | --- |
|  | Pearson’s r | p | Pearson’s r | p< |
| Number of falls | -0.315 | 0.06 | 0.35 | 0.05 |
| Berg Balance Scale | 0.492 | <0.01 | -0.66 | 0.01 |
| Short Physical Performance Battery | -0.081 | 0.53 | -0.60 | 0.01 |
| Activities-Specific Balance Confidence | 0.202 | 0.17 | -0.52 | 0.01 |
| Timed Up and Go Test | -0.330 | 0.06 | 0.66 | 0.01 |
| Community Balance and Mobility Scale | 0.036 | 0.84 | -0.52 | 0.01 |
| Dynamic Gait Index | 0.275 | 0.12 | -0.58 | 0.01 |
| Functional Reach Test | 0.088 | <0.01 | -0.22 | n.s. |

**PARKINSON’S DISEASE DATASET**

The recalculated EGVI values for individuals with PD are summarized in Table A4. The first section displays the values for the total cohort (n=100), whereas the lower sections indicate the number of individuals with normal variability (EGVI=100), lower variability (EGVI < 100) and higher variability (EGVI > 100) than the reference mean, and the respective EGVI means and standard deviations. This is done for overall EGVI (mean value of the left and right side) and EGVI of the most affected side. Values for sub-groups Hoehn & Yahr 2 (mild PD) and 3 (moderate PD) are also shown.

**Table A4.**

EGVI in individuals with Parkinson’s Disease (means and standard deviations)

|  | PD cohort total | Hoehn & Yahr 2 | Hoehn & Yahr 3 | p-value |
| --- | --- | --- | --- | --- |
| Number of participants | 100 | 44 | 56 |  |
| Sex (male/female) | 56/44 | 25/19 | 32/24 |  |
| GVI overall | 97.5 (11.7) | 98.1(9.9) | 97.1 (12.9) | 0.66 |
| EGVI overall | 107.1 (10.3) | 105.0 (9.1) | 108.7 (11.0) | 0.07 |
| GVI most affected | 94.5 (10.6) | 95.1 (9.1) | 94.1 (11.6) | 0.67 |
| EGVI most affected | 108.0 (11.2) | 105.9 (10.4) | 109.7 (11.6) | 0.09 |
| Number with EGVI = 100 | 35 | 14 | 21 |  |
| Number with EGVI < 100 | 12 | 8 | 4 |  |
| EGVI (low variability) overall | 96.3 (2.8) | 95.5 (3.1) | 97.9 (0.7) | 0.18 |
| EGVI (low variability) most affected | 94.7 (3.5) | 94.0 (4.1) | 96.1 (1.5) | 0.36 |
| Number with EGVI > 100 | 53 | 22 | 31 |  |
| EGVI (high variability) overall | 114.2 (9.4) | 111.7 (8.2) | 116.1 (9.9) | 0.08 |
| EGVI (high variability) most affected | 116.3 (9.1) | 113.9 (8.5) | 118.0 (9.2) | 0.10 |

Abbreviations: PD = Parkinson’s Disease; EGVI = Enhanced Gait Variability Index. Significant differences between H&Y 2 and 3 were explored using independent samples t-test (2-tailed). Significance level was set at 0.05.

The strength of all correlations were interpreted according to Munro (Domholdt, E., *Rehabilitation Research: Principles and Applications*. 3rd edition ed. 2003. p.358); <0.25= little if any correlation; 0.25=low correlation; 0.50= moderate correlation; 0.70=high correlation; 0.90=very high correlation. A low correlation was found between the Mini-BESTest and EGVI (high) (overall EGVI: r= 0.49, p<0.001; EGVI most affected side: r= 0.45, p<0.001) as well as between the 1/TUG and EGVI (high) (overall EGVI: r= 0.42, p=0.002, EGVI most affected side: r= 0.41, p=0.002) (Table A5).

**Table A5.**

Associations between dynamic balance and mobility and the GVI and the EGVI.

|  |  | GVI | | EGVI ^(all)^ | | EGVI ^(high)^ | |
| --- | --- | --- | --- | --- | --- | --- | --- |
|  |  | *r* | *p* | *r* | *p* | *r* | *p* |
| MiniBESTest | Overall | 0.33 | <0.01 | -0.50 | <0.01 | -0.49 | <0.01 |
|  | Most affected | 0.29 | <0.01 | -0.47 | <0.01 | -0.45 | <0.01 |
| 1/TUG | Overall | 0.42 | <0.01 | -0.55 | <0.01 | -0.42 | <0.01 |
|  | Most affected | 0.42 | <0.01 | -0.56 | <0.01 | -0.41 | <0.01 |

The EGVI ^(all)^ = total PD cohort, n = 100. EGVI ^(high)^ = subjects with high gait variability, n = 53. r = the Pearson’s correlation coefficient with corresponding p-value.

The discriminatory ability of the EGVI (Table A6), with regards to the ability to distinguish between H&Y 2 and 3 was investigated using ROC curve analysis (Figure A3) in those subjects that showed higher gait variability than the reference mean.

The ROC curve sensitivity against 1-specificity was plotted for the H&Y score, where sensitivity was defined as the number of individuals correctly identified by the index as classified as H&Y 2. We defined specificity as the number of individuals correctly identified by the index as being classified as H&Y 3. Equal importance of high sensitivity and specificity was assumed (Farrar et al, 2001). The area under the ROC is interpreted as the probability of correctly identifying the individuals classified as H&Y 2 from randomly selected pairs of individuals classified as H&Y 2 and 3. The area ranges from 0.5 (no accuracy in discriminating H&Y 2 from 3) to 1.0 (perfect accuracy). The strength of discrimination was interpreted according to Hosmer and Lemeshow (Veierød, M.B., Lydersen, S., Laake, P., *Medical Statistics in clinical and epidemiological research*. 1^st^ ed. 2012: Gyldendal akademisk. p.473); 0.5=no discrimination; 0.7=acceptable; 0.8=excellent; 0.9=outstanding.

**Table A6.**

ROC analysis: Area Under the Curve for GVI and EGVI^(high)^

|  | AUC (GVI) | *p* | AUC (EGVI ^(high)^) | *p* |
| --- | --- | --- | --- | --- |
| Overall | 0.529 | 0.62  0.57 | 0.630 | 0.11 |
| Most affected side | 0.533 |  | 0.633 | 0.10 |

EGVI ^(high)^ = subjects with high gait variability, n=53.

(A) (B)


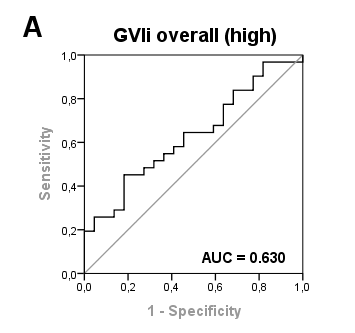

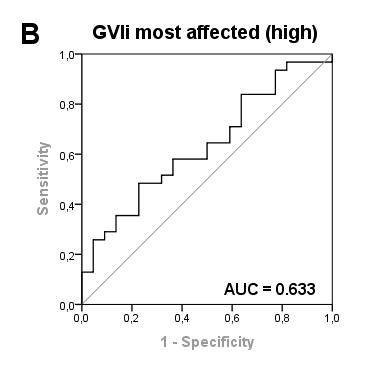


**Figure A3.** Sensitivity and specificity of the overall EGVI^(high)^ (A) and the most affected side EGVI^(high)^ (B). The EGVI for those with higher gait variability than the reference mean is included in the analysis (n=53).
